# Dietary Approaches to Stop Hypertension Diet Pattern and Metabolic Syndrome Prevalence Among U.S. Young Adults: Cross‐Sectional Study From the NHANES 2011–2018

**DOI:** 10.1002/hsr2.73194

**Published:** 2026-10-07

**Authors:** Mary R. Lee, Claire E. Branley, Jung Ae Lee, Bill M. Jesdale, Sarah N. Forrester

**Affiliations:** ^1^ Department of Population and Quantitative Health Sciences University of Massachusetts Chan Medical School Worcester Massachusetts USA

**Keywords:** central obesity, DASH diet, dietary patterns, metabolic syndrome, NHANES, young adults

## Abstract

**Background and Aims:**

Metabolic syndrome (MetS) is a group of interrelated risk factors associated with elevated risk of cardiovascular disease and Diabetes Mellitus. The United States (U.S.) prevalence of MetS is rising, with concerning trends among young adults necessitating updated prevalence estimates of MetS using the most recent MetS criteria. This study describes MetS prevalence and its association with Dietary Approaches to Stop Hypertension (DASH) diet patterns among U.S. adults aged 18–29 years, using National Health and Nutrition Examination Survey (NHANES) data.

**Methods:**

Using cross‐sectional data from 2011 to 2018 NHANES, a nationally representative sample of U.S. adults (analytic sample *n* = 3332 young adults, weighted *n* = 37,981,063) was included. Multivariable weighted log‐binomial models estimated the adjusted prevalence ratio (aPR) for MetS and its five individual components comparing those with high versus low DASH scores, adjusting for socioeconomic and lifestyle factors.

**Results:**

Average participant age was 24 years and 51.8% were women. Over one‐third (35.6%) of young adults met MetS criteria. Most participants (87.5%) did not meet DASH nutrient targets intake amounts. High fasting glucose was the most prevalent MetS component (66.4%). In this sample, while high DASH scores were not associated with MetS presence (aPR: 0.86, 95% CI: 0.72–1.04), they were associated with lower prevalence of central obesity (waist circumference), a component of MetS (aPR: 0.79, 95% CI: 0.64–0.98).

**Conclusion:**

Despite low overall adherence to the DASH diet, higher adherence was linked to lower prevalence of MetS central obesity. Findings support targeted nutrition strategies in early adulthood to help reduce long‐term cardiometabolic risk.

## 1 Introduction

Metabolic syndrome (MetS) is characterized by the clustering of hypertension, obesity, dyslipidemia, and elevated blood glucose, and is associated with an elevated risk of cardiovascular disease (CVD) and type 2 diabetes [1, 2]. The prevalence of MetS has risen sharply in the United States (U.S.), increasing from one‐in‐four to more than one‐in‐three U.S. adults over the past three decades [3]. This trend poses a public health challenge, as MetS is linked to increased risk of disability [4], obesity‐related conditions, and premature deaths from CVD [5, 6]. While older adults often experience MetS at higher rates [4, 5, 7], prevalence of MetS increases from nearly 4% in adolescence to approximately 10% by young adulthood [3, 8, 9].

Young adulthood represents a critical period for establishing lasting health behaviors, including dietary patterns [10]. During this life stage, individuals often develop long‐term social and dietary habits that can shape metabolic health trajectories [11]. However, young adults often have suboptimal diets, influenced by factors such as socioeconomic status, education, and food environment, which further exacerbate metabolic risk [10, 11].

The Dietary Approaches to Stop Hypertension (DASH) emphasizes higher intake of fruits, vegetables, and low‐fat dairy while minimizing meats and sweets [12, 13]. It incorporates a diet with abundant fiber, minerals, and vitamins, while limiting quantities of total and saturated fat, dietary cholesterol, and sodium [11]. Originally designed to manage hypertension [13], DASH also confers metabolic advantages, including the prevention of obesity and regulation of blood serum and glucose levels [14, 15]. Although numerous studies have shown an association between MetS and low diet quality in older adults [12, 16], few have examined the DASH dietary patterns and MetS in young adults [14, 17].

Furthermore, to our knowledge, no prior study has estimated the prevalence of MetS among U.S. young adults using the harmonized definition of MetS proposed in the joint interim statement from the International Diabetes Federation (IDF) Task Force and partner organizations [1]. This unified definition incorporates country‐ and population‐specific criteria for MetS components, enabling more consistent prevalence estimates across diverse populations.

Using nationally representative data from the 2011–2018 National Health and Nutrition Examination Survey (NHANES), this study provides contemporary prevalence estimates of MetS among U.S. young adults aged 18–29 years. A secondary objective was to examine whether DASH diet adherence in young adulthood was associated with MetS and its individual components. We hypothesized that young adults with higher DASH scores would be less likely to meet MetS criteria.

## 2 Methods

The UMass Chan Medical School Institutional Review Board considered this study exempt as publicly available de‐identified data were used.

### 2.1 Study Design and Participants

We used cross‐sectional data from the 2011–2018 NHANES, a biennial survey conducted by the National Center for Health Statistics. NHANES provides a comprehensive assessment of the health status of approximately 5000 community‐dwelling residents each year in the U.S. NHANES has seen reduced participation rates between 2011 and 2018, ranging from 79% to 54% (unweighted) [18]. To mitigate the potential effects of response bias due to the lower response rate in the 2017–2018 NHANES cycle, NHANES employed additional weighting adjustments to this cycle [19]. NHANES is a series of nationally representative surveys that used multi‐stage and stratified sampling designs [20]. Trained staff conducted an interview and, for a subset of participants, also conducted a physical examination and laboratory tests in mobile examination centers (MEC) [19]. For the present study, we combined the 2011–2012, 2013–2014, 2015–2016, and 2017–2018 NHANES waves where anthropometric, physiological, laboratory measurements, and questionnaire data were used to measure MetS, and total nutrient intake and dietary data derived from a 24‐h in‐person dietary recall interview (Day 1). Day 2 dietary interview data, conducted 3–10 days post Day 1 data collection via telephone, were not used in this study due to lower study participation rates [18, 19]. Prior survey cycles were not used in the present analysis due to oversampling previously underrepresented subgroups of persons identifying as non‐Hispanic Asian, Hispanic, non‐Hispanic Black, and non‐Hispanic White at or below 130% of the poverty level and 80 years of age or older [19]. Survey cycles following 2018 were not used in this study due to the COVID‐19 pandemic, which led to the suspension of field operations and collection of participant data [21].

A total of 39,156 participants from 2011 to 2018 NHANES were enumerated in the initial sample. Of these, 4960 were young adults 18–29 years of age. We included participants who had participated in the Day 1, 24‐h dietary recall interview and who had complete total nutrient data (*n *= 4341). We excluded participants who reported being pregnant (*n *= 128), had three or more missing MetS components (*n *= 323), or had missing or incomplete covariate data (*n *= 558). The final study sample consisted of 3332 young adults, representing 37,981,063 young American adults (Figure 1).

**Figure 1:**
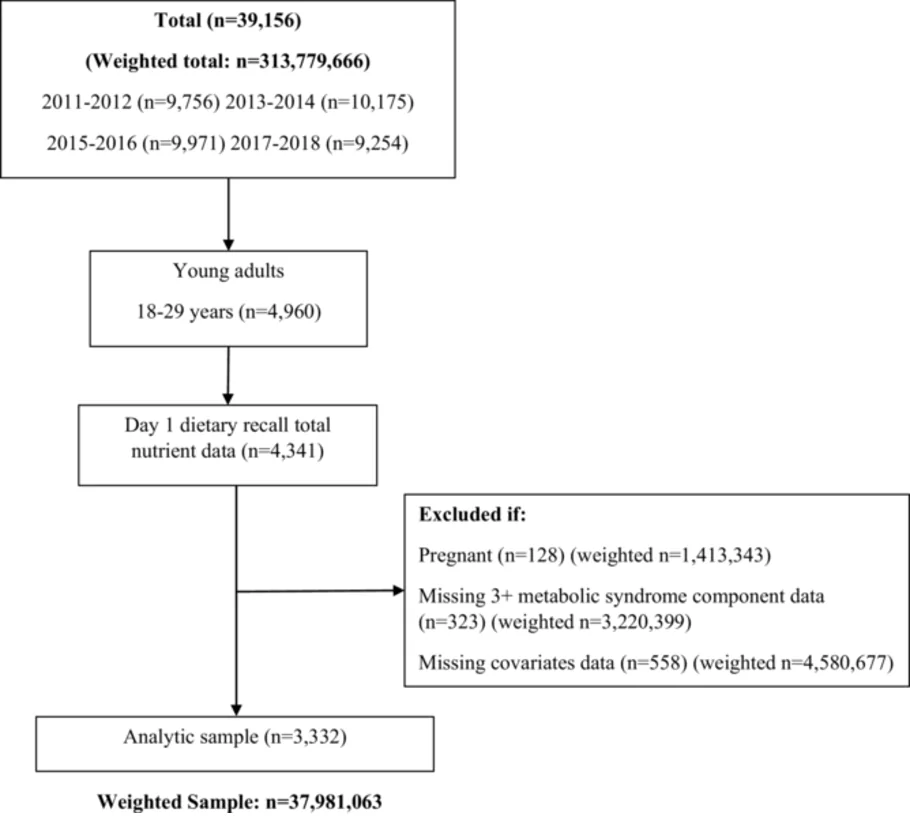
Flow chart of participant selection for the analytical sample from NHANES 2011–2018. Legend: The flow chart summarizes inclusion and exclusion criteria used to derive the final analytic sample of U.S. young adults aged 18–29 years. Participants were excluded if they were pregnant, missing ≥ 3 metabolic syndrome component variables, or missing covariate data. Weighted totals reflect NHANES survey sampling weights. NHANES, national health and nutrition examination survey.

### 2.2 Metabolic Syndrome

MetS was defined using the harmonized criteria from the Joint Interim Statement of the IDF Task Force on Epidemiology and Prevention, in collaboration with other major organizations [1]. Participants were classified as having MetS if they met three or more of the following five components: (1) waist circumference of ≥ 88 cm in females or ≥ 102 cm in males, (2) serum triglyceride level of ≥ 150 mg/dL or use of medication to treat high triglycerides, (3) high‐density serum lipoprotein cholesterol (HDL‐C) levels of < 50 mg/dL in females or < 40 mg/dL in males, or use of medication to treat reduced HDL‐C, (4) blood pressure (BP) of ≥ 130 mmHg systolic or ≥ 85 mmHg diastolic, or use of medication to treat high BP, and (5) fasting blood sugar levels of ≥ 110 mg/dL, or use of medication to treat elevated serum glucose values. Laboratory values were obtained from NHANES MEC data. When laboratory measures were unavailable, self‐reported medication use from questionnaire data was used to assess component status. Systolic and diastolic BP were averaged across three total readings. MetS was coded as a binary variable, as being present if the participant had three or more MetS components (present vs. absent). Each individual component was similarly coded as a binary variable, indicating whether a participant met the specified criterion.

### 2.3 DASH Dietary Patterns

Total nutrient intake was assessed using data from the Day 1 dietary recall interview, a 24‐h recall conducted in the NHANES MEC [22]. Based on these data, nine total nutrient components from the DASH diet were evaluated: macronutrients (total fat, saturated fat, and protein) and micronutrient components (fiber, calcium, magnesium, potassium, and sodium) and cholesterol [23]. Following the DASH diet index methodology [23], all nutrient values were normalized based on total caloric intake (energy‐adjusted). Macronutrients were expressed as the percentage of total caloric intake [24], while micronutrients were expressed per 1000 kilocalories (kcal). Each participant's intake was compared with DASH target and intermediate target levels, as shown in Table 1. Scoring following established protocols [23, 25], participants received 1 point for meeting a nutrient target, 0.5 points for meeting the intermediate target, and 0 points if their intake was above or below the intermediate range, depending on the nutrient. Scores across the nine‐nutrient component were summed to yield a total DASH score (ranging from 0 to 9), with higher scores indicating closer alignment with DASH dietary nutrient recommendations. For consistency with prior studies and to aid interpretation, total DASH scores were dichotomized at 4.5. Scores ≥ 4.5 indicated adherence to the DASH dietary pattern, reflecting achievement of roughly half of the DASH nutrient targets, scores < 4.5 indicated non‐adherence [23, 25].

**Table 1:** DASH diet target, intermediate, and above or below intermediate nutrient amount thresholds for DASH score.^a^

Nutrient	DASH target nutrient amount	DASH intermediate nutrient amount	Above or below DASH intermediate nutrient amount
Total fat	27% of kcal	32% of kcal	> 11% of energy
Saturated fat	6% of kcal	11% of kcal	> 32% of energy
Protein	18% of kcal	16.5% of kcal	< 16.5% of energy
Fiber	14.8 g/1000 kcal	9.5 g/1000 kcal	< 9.5 g/1000 kcal
Cholesterol	71.4 mg/1000 kcal	107.1 mg/1000 kcal	> 107.1 mg/1000 kcal
Magnesium	238 mg/1000 kcal	158 mg/1000 kcal	< 158 mg/1000 kcal
Calcium	590 mg/1000 kcal	402 mg/1000 kcal	< 402 mg/1000 kcal
Potassium	2238 mg/1000 kcal	1534 mg/1000 kcal	< 1534 mg/1000 kcal
Sodium	1143 mg/1000 kcal	1286 mg/1000 kcal	> 1286 mg/1000 kcal

Abbreviations: DASH, dietary approaches to stop hypertension; g, grams; kcal, kilocalories; mg, milligrams.

^a^ Values are weighted percentages of target and intermediate DASH scores of individual total nutrient component intake amount per 1000 kilocalories (kcal). DASH target, intermediate, and above or below intermediate scores were based on DASH diet indexing methodology [23].

### 2.4 Covariates

Potential confounding factors known to be associated with the DASH diet and/or MetS were identified based on literature review and a priori assumptions. Demographic, socioeconomic characteristics, and lifestyle variables were considered. This includes participant age, sex (female/male), education (less than high school, high school diploma/GED, some college or Associate's degree, college graduate or higher), and race/ethnicity (non‐Hispanic White, non‐Hispanic Black, Hispanic, non‐Hispanic Asian, other and multi‐racial) as a proxy for racialized life experience in the U.S [26]. Occupational status was categorized as employed, unemployed, student, or other, other categories included respondents unable to work at the time of participation due to health issues, disability, and care for family members. Smoking status was determined by the question, “Have you smoked at least 100 cigarettes in your whole life?” (no/yes). Food security was based on the NHANES “adult food security category for last 12 months”, derived from 10 questions assessing household and individual food security. Questions addressed whether a person could not afford to eat balanced meals or had to reduce meal sizes or skip meals due to lack of money for food. Responses were categorized as high (0 affirmative responses), marginal (1–2 affirmative responses), or low (3–10 affirmative responses). Income‐to‐poverty ratio (IPR) was calculated by dividing household income by the poverty threshold specific to household composition, year, and state residency [27]. IPR was categorized as not impoverished (IPR ≥ 2.00), near poverty (IPR 1.00–1.99), or impoverished (IPR < 1.00) [27]. Self‐reported physical activity was determined from household interview questions asking participants to recall activities performed in the past 30 days. Physical activity levels were converted to metabolic equivalent (MET) minutes and classified into active or inactive, using 150 min/week physical activity recommendation as the threshold. Both total physical activity and total recreational physical activity were assessed.

### 2.5 Statistical Analysis

All analyses incorporated NHANES survey weights (e.g., MEC examination weights, Day 1 dietary weights) to account for the complex, multistage sampling design. Because four 2‐year cycles (2011–2018) were combined, weights were divided by four in accordance with NHANES analytic guidelines [22]. The NHANES‐provided weights incorporate adjustments for differential probabilities of selection, nonresponse, and post‐stratification to match independent population estimates by age, sex, and race/ethnicity categories [22]. Descriptive characteristics were calculated and described according to the presence of MetS. Categorical variables are presented as frequencies (percentages) and continuous variables are presented as means with standard deviation (SD). To describe dietary patterns, total DASH diet target, intermediate, and above or below intermediate scores were calculated. Prespecified primary analyses examined the relationship between the dichotomized DASH score and MetS (and its five individual components) using multivariable weighted log‐binomial models to estimate unadjusted and adjusted prevalence ratios (aPR) with 95% confidence intervals (CI). Multivariable adjusted models included sex, race/ethnicity, age, and recreational physical activity level. Covariate inclusion in final models was guided by prior conceptual and empirical considerations, with emphasis on maintaining model parsimony. Smoking status, food security, and IPR were considered but not retained in primary models because they are plausibly on the causal pathway between dietary patterns and MetS or could introduce overadjustment. Accordingly, final models were specified to estimate associations while minimizing adjustment for potential mediators. Sequentially minimally adjusted models for exploratory analyses were conducted to evaluate how adjustment for covariates affected the estimates using multivariable weighted log‐binomial regression. These models first controlled for demographic characteristics (age, sex, and race/ethnicity) and then for a lifestyle factor (physical activity). Sensitivity analyses were performed using the continuous total DASH score (range 0–9) to assess the robustness of findings across alternative exposure definitions using multivariable weighted log‐binomial models similar to the primary analyses. All inferential statistical tests were two‐sided, and a *p* value < 0.05 was considered statistically significant. All statistical analyses were conducted using Stata MP 18.0 (StataCorp, College Station, TX, USA).

## 3 Results

The mean age of participants was 24 years (SD: 3.8), and 51.8% were female (Table 2). Most were non‐Hispanic White, had completed some college, were employed, reported high food security, and engaged in regular physical activity. The average DASH score was 2.7 (SD: 1.7) for individuals who met criteria for MetS, and 2.9 (SD: 1.8) for those who did not. Table 3 presents the proportion of young adults meeting DASH nutrient intake targets, intermediate, and above or below intermediate amounts. The lowest DASH target adherence rates were observed for sodium, potassium, magnesium, saturated fat, and fiber, while protein intake had the highest adherence rates, with 86.2% (95% CI: 84.5%–87.7%) meeting target goals. Most participants (87.5%) did not meet all DASH intermediate nutrient goals, and 0.2% met none.

**Table 2:** Sample characteristics of U.S. young adults (18–29 years) according to the presence of metabolic syndrome, 2011–2018 NHANES (*N* = 37,981,063).^a^

	Metabolic syndrome present (weighted)	Metabolic syndrome absent (weighted)
Unweighted sample, no.	1185	2147
Weighted, no.	13,509,864	24,471,199
Age (mean years, SD)	24.3 (3.8)	23.5 (5.5)
Female (%)	51.8	45.9
Race/ethnicity (%)^b^		
Non‐Hispanic White	55.6	59.0
Non‐Hispanic Black	12.5	12.2
Hispanic	22.7	18.9
Non‐Hispanic Asian	4.1	6.4
Other and multi‐racial	5.1	3.5
Highest level of education (%)		
College graduate or higher	18.3	25.8
Some college, including AA	33.9	33.6
High school diploma or GED	33.7	28.6
Less than high school diploma	14.1	12.0
Occupational status (%)		
Employed	71.4	74.6
Unemployed	9.7	7.3
Student	6.7	9.8
Other^c^	12.2	8.3
Smoked cigarettes (%)	35.6	30.3
Food security (%)^d^		
High security	58.7	67.6
Marginal security	16.4	13.1
Low security	24.9	19.3
Income‐to‐poverty ratio (%)^e^		
Not impoverished	50.7	53.0
Near poverty	24.8	24.1
Impoverished	24.5	22.9
Physical activity level (MET‐min/wk) (%)^f^		
Total physical activity		
Active (≥ 600)	77.6	81.8
Inactive (< 600)	22.4	18.2
Total recreational physical activity		
Active (≥ 600)	47.6	56.9
Inactive (< 600)	52.4	43.4
DASH score (mean, SD)	2.7 (1.7)	2.9 (1.8)

Abbreviations: DASH, dietary approaches to stop hypertension; IPR, income‐to‐poverty ratio; NHANES, national health and nutrition examination survey; MET, metabolic equivalent; min, minutes; SD, standard deviation; wk, week.

^a^ Values are the weighted mean (SD) and percentages. Unweighted, n = 3,332.

^b^ Hispanic race/ethnicity includes Mexican American and other Hispanic origin. Other and multi‐racial race/ethnicity includes other and non‐Hispanic multi‐racial.

^c^ Other occupation status includes individuals unable to work at time of NHANES participation due to indicated health concerns, disability, or caring for family member(s).

^d^ Food security categorized through the U.S. Household Food Security Survey Module developed by the U.S. Department of Agriculture. High=0, marginal=1‐2, low=3‐10.

^e^ IPR is calculated by dividing household income by the U.S. Census Bureau poverty threshold, adjusted for household size, year, and state of residence. Categories: Not Impoverished (IPR ≥ 2.00), Near Poverty (IPR 1.00‐1.99), Impoverished (IPR < 1.00) [27].

^f^ Physical activity measured as 600 MET‐min/wk, equivalent to 150 min/wk, moderate‐intensity activity rated at 4 METS and vigorous‐intensity activity rated at 8 METS per NHANES guidelines. Total physical activity represents activity for work and transportation.

**Table 3:** Proportion meeting DASH diet nutrient targets, intermediate, and above or below intake of DASH nutrient components, NHANES 2011–2018 (*N* = 37,981,063)^a^, ^b^, ^c^.

Nutrient	U.S. Young Adults		
	DASH target intake (%, 95% CI)	DASH intermediate intake (%, 95% CI)	Above or below intermediate intake (%, 95% CI)
Total fat	17.5 (15.7–19.4)	19.1 (17.1–21.1)	63.4 (61.0–65.8)
Saturated fat	9.3 (8.2–10.5)	37.9 (35.7–40.0)	52.8 (50.3–55.2)
Protein	86.2 (84.5–87.7)	4.4 (3.4–5.6)	9.4 (7.9–10.9)
Fiber	5.8 (4.8–7.0)	17.1 (15.2–19.3)	77.1 (74.7–79.3)
Cholesterol	24.0 (22.0–26.0)	23.7 (22.0–25.5)	52.3 (49.9–54.7)
Magnesium	5.6 (4.5–6.7)	22.3 (20.1–24.7)	72.1 (69.6–74.4)
Calcium	25.1 (22.8–27.4)	32.0 (30.2–33.9)	42.9 (40.5–45.1)
Potassium	2.2 (1.6–3.1)	14.5 (12.9–16.2)	83.3 (81.3–85.0)
Sodium	10.9 (9.3–12.7)	7.8 (6.5–9.3)	81.3 (78.6–83.6)

Abbreviations: CI, confidence intervals; DASH, dietary approaches to stop hypertension; NHANES, national health and nutrition examination survey.

^a^ Values are weighted percentages of DASH scores of individual total nutrient component intake amount per 1000 kilocalories. DASH target, intermediate, and above and below intermediate scores were based on DASH Diet indexing methodology [23].

^b^ Unweighted, *n* = 3332.

^c^ Among the weighted sample, 0.2% did not meet any intermediate or target nutrient amounts, 87.5% met only one intermediate nutrient amount, 11.9% met all but one intermediate amount, 0.3% met all intermediate amounts and at least one target nutrient amount, 0.1% met all target DASH nutrient amounts.

The weighted prevalence of MetS was 35.6% (95% CI: 32.9%–38.3%), whereas the prevalence of individual MetS components ranged from 12.5% to 66.4% (Table 4). High fasting glucose was the most common component (66.4%), whereas high BP was the least common (12.5%). High DASH adherence was associated with lower prevalence of central obesity (waist circumference) in both unadjusted and adjusted models (aPR: 0.79, 95% CI: 0.64–0.98). Estimates for other MetS components, including high triglycerides, high BP, low HDL‐C, and high fasting glucose, were closer to the null. After adjustment for sex, race/ethnicity, age, and recreational physical activity, high DASH scores were not significantly associated with overall MetS prevalence (aPR: 0.86, 95% CI: 0.72–1.04).

**Table 4:** Association between DASH index score and individuals who met criteria for metabolic syndrome and components among young U.S. adults, NHANES 2011–2018 (*N* = 37,981,063).^a^

	Weighted, Prevalence^b^	Unadjusted	Adjusted^c^
	(%, 95% CI)	Prevalence ratio (95% CI)	Prevalence ratio (95% CI)
Metabolic syndrome	35.6 (32.9–38.3)	0.87 (0.72–1.03)	0.86 (0.72–1.04)
Metabolic syndrome components			
Wide waist circumference	37.8 (34.9–40.7)	0.79 (0.64–0.99)	0.79 (0.64–0.98)
High triglycerides	58.0 (55.8–60.2)	1.07 (0.95–1.21)	1.05 (0.93–1.18)
High blood pressure or Rx	12.5 (11.3–13.7)	0.99 (0.68–1.44)	1.04 (0.72–1.51)
Low HDL‐C or Rx	29.1 (26.4–31.9)	0.88 (0.71–1.08)	0.88 (0.72–1.09)
High fasting glucose or Rx	66.4 (63.9–68.7)	0.99 (0.91–1.09)	1.00 (0.92–1.09)

Abbreviations: CI, confidence intervals; DASH, dietary approaches to stop hypertension; HDL‐C, high‐density lipoprotein cholesterol; NHANES, national health and nutrition examination survey; Rx, prescription.

^a^ Unweighted sample, *n* = 3332.

^b^ Weighted prevalence derived from DASH exposure variable determined based on DASH diet index scoring [23]. Higher DASH diet includes summed total nutrient scores ≥ 4.5, and lower DASH diet summed score < 4.5. Based on the Joint Statement on Metabolic Syndrome criteria [1]. Waist circumference is based on recommended waist circumference thresholds for abdominal obesity by the American Heart Association and National Heart, Lung, and Blood Institute [1, 28].

^c^ Adjusted models were adjusted for sex, race/ethnicity, age, and recreational physical activity.

In minimally adjusted models, waist circumference remained associated with DASH adherence in demographic‐adjusted models (aPR: 0.76, 95% CI: 0.61–0.94), although the estimate was attenuated after additional adjustment for the lifestyle factor of physical activity (Supporting Information S1: Table S1). However, higher DASH scores were not associated with materially different MetS prevalence in either the demographic‐adjusted (aPR: 0.84, 95% CI: 0.70–1.00) or lifestyle‐adjusted models (aPR: 0.90, 95% CI: 0.75–1.08).

Sensitivity analyses using the continuous total DASH score yielded similar findings consistent with the primary dichotomized analysis (Supporting Information S1: Table S2). Higher DASH scores were not associated with MetS prevalence overall (aPR: 0.97, 95% CI: 0.92–1.01). However, each one‐point increase in DASH score was associated with a lower prevalence of waist circumference (aPR: 0.94, 95% CI: 0.89–0.98). Estimates for the remaining MetS components were centered near the null.

## 4 Discussion

This study analyzed nationally representative data from NHANES 2011–2018 to examine MetS prevalence and whether dietary patterns aligned with the DASH diet were associated with MetS and its individual components among young U.S. adults. Weighted prevalence estimates indicated that 3 out of 10 young adults met the criteria for MetS. Most participants did not meet the DASH diet's recommended nutrient targets, with many consuming amounts above or below the intermediate thresholds. Sodium, potassium, magnesium, and fiber had the highest proportions of young adults not meeting DASH target or intermediate amounts. While high DASH diet adherence was not associated with overall MetS prevalence, it was associated with lower prevalence of central obesity, as measured by waist circumference.

### 4.1 Metabolic Syndrome Prevalence

Approximately one‐third of young adults met the criteria for MetS in our study, a prevalence that appears higher than the 10%–20% range reported in prior literature [3, 8, 9]. However, this estimate reflects the use of the harmonized Joint Interim Statement definition, including the incorporation of medication use, and should not be directly compared with estimates from differing criteria. Several factors likely explain this difference. First, prior studies have used varying definitions of MetS, most commonly the IDF criteria or the National Cholesterol Education Program Adult Treatment Panel III (NCEP/ATP III) criteria, which differ in their thresholds for waist circumference and in how they account for medication use [8, 9, 28, 29]. The harmonized definition from the Joint Interim Statement, used in the present study, standardizes component thresholds across major global organizations, treats waist circumference as one of five possible components (rather than a required feature), and includes current medication use for most conditions in determining component status [1]. Second, much of the existing literature draws on different NHANES cycles or focuses on older or adolescent populations. For example, studies of adults aged ≥ 20 years have reported MetS prevalence near 33% [7, 9], whereas estimates among adolescents are closer to 5% [8, 30]. Finally, secular trends in key factors, such as type 2 diabetes, hypertension, and abdominal obesity [15, 31], may also contribute to higher prevalence in most recent data. These methodological and population differences, particularly the use of a harmonized definition that treats waist circumference as one of five non‐mandatory components and incorporates medication use, likely contribute to the higher prevalence observed in our study and limit direct comparability with studies that use a different MetS definition. Our study adds to this literature by providing an updated, nationally representative estimate of the MetS prevalence among young U.S. adults aged 18–29 years using the harmonized global definition, capturing both measured biomarkers and medication use.

### 4.2 Dietary Findings

Consistent with prior research, our study found that young U.S. adults frequently exceed dietary recommendations for several nutrients and fall short on others [24]. Average sodium intake was approximately 3430 mg/day (based on a 2000‐calorie diet), with 81% of participants exceeding the Dietary Guidelines for Americans' recommended maximum of 2300 mg/day [24]. This is comparable to national estimates for U.S. adults (approximately 3400 mg/day) [24, 32] and underscores the persistent challenge of reducing sodium intake, even among regularly physically active individuals [33].

We also found that potassium, magnesium, fiber, and sodium were nutrients most often consumed above or below the recommended DASH target or intermediate thresholds among young adults, contributing to low overall DASH scores. Similar patterns have been observed in early NHANES cycles. For example, analyses of 2003–2012 data demonstrated similar DASH scores ranging from 1.48 to 2.0 among children and adolescents [32, 34], while NHANES 2004–2006 data for adults aged ≥ 20 years reported mean DASH scores of 1.95–2.02 using a nutrient‐based DASH scoring method similar to ours [33]. In other national analyses of adults with hypertension, mean scores were closer to 2.9, suggesting slightly higher adherence in higher‐risk groups [23]. While that study also used a nutrient‐based scoring method and included younger adults (aged 20–39 years) as a reference group [23], differences in age distribution and health status limit direct comparability to our sample. Collectively, these NHANES‐based studies indicate that DASH adherence remains low across both general and high‐risk adult populations in the U.S.

In our analysis, high DASH scores were not associated with a materially different prevalence of MetS or most of its individual components. However, a modest inverse association was observed for central obesity (waist circumference), consistent with evidence that DASH adherence may be related to abdominal adiposity even when associations with overall MetS are limited [23, 25]. Variability in observed associations across studies may reflect differences in how DASH adherence is conceptualized and operationalized. Some studies have used food group‐based measures (e.g., servings of fruits, vegetables, or whole grains) operationalized as continuous DASH scores [12, 13, 14], whereas our approach used nutrient‐based intake targets operationalized primarily as a dichotomized DASH variable, with continuous DASH scores examined in sensitivity analyses. These differing approaches may capture dietary quality through a different lens and influence the ability to detect dose‐response relationships. Overall, these findings highlight the ongoing challenge of achieving adequate nutrient intake in young adulthood and the complexity of linking dietary patterns to multifactorial outcomes like MetS.

### 4.3 Barriers to Dietary Guidelines Adherence

Low DASH adherence in our sample likely reflects a combination of individual and environmental barriers. At an individual level, factors such as nutrient knowledge, misinterpretation of dietary advice, financial constraints, and low motivation to change dietary patterns can hinder healthier choices [31]. At a neighborhood level, food environment plays a critical role, limited access to affordable, healthy foods and the ubiquity of inexpensive, energy‐dense options may reinforce suboptimal dietary habits, particularly in certain geographic or socioeconomic contexts [31].

These findings may have important public health and clinical implications for young adults, a population often perceived to be at relatively low cardiometabolic risk despite the prevalence of MetS observed in this study. Interventions aimed at improving access to affordable nutrient‐dense foods, strengthening nutrition education, and promoting healthier dietary behaviors in early adulthood may help address modifiable metabolic risk factors before progression to chronic disease. In clinical settings, routine screening and counseling related to dietary quality and metabolic health may also help identify individuals at elevated cardiometabolic risk who may otherwise go unrecognized. Future research should examine how these individual‐ and neighborhood‐level determinants may shape dietary patterns and MetS prevalence in early adulthood.

### 4.4 Strengths and Limitations

A major strength of this study is the use of nationally representative NHANES data collected with standardized protocols by trained personnel, which supports the generalizability and validity of the findings. Selection bias is minimized through the continuous, randomized sampling design that covers diverse regions and population subgroups across the U.S.

Several limitations should be considered when interpreting these results. First, dietary intake was assessed using a single 24‐h recall, which captures a snapshot of participants' eating patterns rather than their usual dietary intake. Although single recalls have been used to estimate DASH adherence in large population studies [23, 25, 31], they are subject to recall error and misclassification bias [35]. Because dietary intake was measured similarly across participants regardless of MetS status, any resulting misclassification would likely be nondifferential and may have attenuated associations toward the null. In addition, advance knowledge of the dietary interview may have led to underreporting of total energy and nutrient intakes [18, 32]. Second, because this study measured adherence to a DASH diet pattern and not adherence following individualized counseling, findings should be interpreted as reflecting alignment with DASH nutrient goals rather than the effects of a prescribed DASH intervention. Additionally, the primary dichotomization of DASH adherence may have reduced variability and limited the ability to detect potential dose–response relationships. Finally, the cross‐sectional design does not capture long‐term dietary behavior, nor can it determine whether dietary patterns preceded the development of MetS or its components. It is also possible that metabolic abnormalities or related health concerns influenced participants' dietary behaviors, such that DASH‐related patterns may reflect responses to metabolic risk rather than antecedents of disease. Despite these limitations, this study provides updated nationally representative estimates of MetS prevalence and DASH‐related patterns among U.S. young adults using a harmonized MetS definition and standardized NHANES assessment.

## 5 Conclusions

This study shows that approximately one‐third of U.S. young adults met the criteria for MetS, and that their dietary patterns fell short of DASH dietary recommendations. While overall DASH adherence was not associated with MetS prevalence, it was associated with lower prevalence of central obesity. These findings should not be interpreted as evidence of temporal relationships between DASH adherence and metabolic health due to the cross‐sectional study design. Further research is needed to explore the mechanisms underlying these relationships, including how individual‐ and neighborhood‐level factors may mediate or modify the relationship between DASH adherence and MetS.

## Author Contributions

**Mary R. Lee:** conceptualization, methodology, formal analysis, project administration, funding acquisition, writing – original draft, visualization, investigation. **Claire E. Branley:** methodology, writing – review and editing, visualization. **Jung Ae Lee:** writing – review and editing, visualization. **Bill M. Jesdale:** visualization, writing – review and editing, methodology. **Sarah N. Forrester:** supervision, conceptualization, resources, writing – review and editing, visualization.

## Ethics Statement

Ethical approval for this study was granted by the ethical review committee of the National Center for Health Statistics. Written consent was received from all respondents prior to questionnaire collection. This study was considered not human subjects research by the Committee for the Protection of Human Subjects in Research at UMass Chan Medical School.

## Conflicts of Interest

The authors declare no conflicts of interest.

## Transparency Statement

The lead author, Mary R. Lee, affirms that this manuscript is an honest, accurate, and transparent account of the study being reported; that no important aspects of the study have been omitted; and that any discrepancies from the study as planned (and, if relevant, registered) have been explained.

## Supporting information


Supporting File

## Data Availability

The data can be accessed from the dataset published in NHANES (https://www.cdc.gov/nchs/nhanes/index.html). The primary data generated or analyzed in this article can be provided upon reasonable request to the corresponding author.
