# Supplementary material for: Dietary Approaches to Stop Hypertension Diet Pattern and Metabolic Syndrome Prevalence Among U.S. Young Adults: Cross‐Sectional Study From the NHANES 2011–2018
Source: Health Sci Rep. 2026 Oct 7;9(10):e73194. doi: 10.1002/hsr2.73194 (PMC13646702; doi:10.1002/hsr2.73194)
Supplement: Supplementary file 1 [file HSR2-9-e73194-s001.docx]

# **Supporting Information**

This file contains additional tables related to the minimally adjusted models and sensitivity analyses referenced in the manuscript: “Dietary Approaches to Stop Hypertension Diet Pattern and Metabolic Syndrome Prevalence Among U.S. Young Adults: Cross-Sectional Study from the NHANES 2011-2018 Approach”.

**Supplementary Table S1.** Minimally adjusted models for the association between DASH index score and those individuals who met criteria for metabolic syndrome and components by demographic and lifestyle factor among young U.S. adults, NHANES 2011-2018 (N=37,981,063).

**Supplementary Table S2.** Sensitivity analysis of the association between total DASH score and individuals who met criteria of metabolic syndrome and components among young U.S. adults, NHANES 2011-2018 (N=37,981,063).

**Supplementary Table S1.**

| Supplementary Table S1. Minimally adjusted models for the association between DASH index score and those individuals who met criteria for metabolic syndrome and components by demographic and lifestyle factor among young U.S. adults, NHANES 2011-2018 (N=37,981,063)† | | | | | | |
| --- | --- | --- | --- | --- | --- | --- |
|  | Unadjusted | | Minimally adjusted for demographics^§^ | | Minimally adjusted for lifestyle^§^ | |
|  | Prevalence Ratio  (95% CI) | p-value | Prevalence Ratio  (95% CI) | p-value | Prevalence Ratio  (95% CI) | p-value |
| Metabolic syndrome | 0.87 (0.72 – 1.03) | 0.150 | 0.84 (0.70 – 1.00) | 0.059 | 0.90 (0.75 – 1.08) | 0.259 |
| Metabolic syndrome components |  |  |  |  |  |  |
| Waist Circumference‡ | 0.79 (0.64 - 0.99) | 0.047 | 0.76 (0.61 – 0.94) | 0.013 | 0.83 (0.66 – 1.03) | 0.098 |
| High Triglycerides | 1.07 (0.95 - 1.21) | 0.262 | 1.05 (0.93 - 1.19) | 0.400 | 1.07 (0.95 - 1.20) | 0.288 |
| High Blood Pressure or Rx | 0.99 (0.68 - 1.44) | 0.942 | 1.02 (0.70 - 1.49) | 0.904 | 1.00 (0.69 - 1.45) | 0.984 |
| Low HDL-C or Rx | 0.88 (0.71 - 1.08) | 0.211 | 0.85 (0.69 - 1.04) | 0.121 | 0.90 (0.73 - 1.12) | 0.350 |
| High Fasting Glucose or Rx | 0.99 (0.91 - 1.09) | 0.946 | 0.99 (0.91 - 1.09) | 0.967 | 0.99 (0.91 - 1.09) | 0.950 |

**Abbreviations:** DASH, Dietary Approaches to Stop Hypertension; HDL-C, High-Density lipoprotein Cholesterol; NHANES, National Health and Nutrition Examination Survey; Rx, prescription.

†Weighted prevalence derived from DASH exposure variable determined based on DASH diet index scoring.^23^ Higher DASH diet includes summed total nutrient scores ≥4.5, and lower DASH diet summed score <4.5. Based on the Joint Statement on Metabolic Syndrome criteria.^1^

‡Waist circumference is based on recommended waist circumference thresholds for abdominal obesity by the American Heart Association and National Heart Lungs and Blood Institute.^1,28^

^§^Weighted minimal models were adjusted sequentially by demographic factor covariates: sex, race/ethnicity, and age, and then for lifestyle factor: recreational physical activity.

**Supplementary Table S2.**

| Supplementary Table S2. Sensitivity analysis of the association between total DASH score and individuals who met criteria of metabolic syndrome and components among young U.S. adults, NHANES 2011-2018 (N=37,981,063).^†^ | | |
| --- | --- | --- |
|  | Unadjusted | Adjusted^¶^ |
|  | Prevalence Ratio (95% CI) | Prevalence Ratio (95% CI) |
| Metabolic syndrome^‡^ | 0.96 (0.92 – 1.02) | 0.97 (0.92 – 1.01) |
| Metabolic syndrome component |  |  |
| Wide Waist Circumference^§^ | 0.93 (0.88 - 0.98) | 0.94 (0.89 - 0.98) |
| High Triglycerides | 1.02 (0.99 - 1.05) | 1.01 (0.98 - 1.04) |
| High Blood Pressure or Rx | 0.93 (0.82 - 1.05) | 0.94 (0.84 - 1.06) |
| Low HDL-C or Rx | 0.98 (0.93 - 1.02) | 0.98 (0.94 - 1.03) |
| High Fasting Glucose or Rx | 0.98 (0.96 - 1.01) | 0.98 (0.96 - 1.01) |

**Abbreviations:** DASH, Dietary Approaches to Stop Hypertension; HDL-C, High-Density lipoprotein Cholesterol; NHANES, National Health and Nutrition Examination Survey; Rx, prescription.

†Weighted prevalence derived from total DASH score (range 0-9) determined based on DASH diet index scoring.^23^

‡Higher total DASH diet score indicates greater adherence to DASH diet target amounts. Metabolic syndrome operationalization was based on the Joint Statement on Metabolic Syndrome criteria.^1^

§Waist circumference is based on recommended waist circumference thresholds for abdominal obesity by the American Heart Association and National Heart Lungs and Blood Institute.^1,28^

^¶^Models were adjusted for sex, race/ethnicity, age, and recreational physical activity.
